# Parameter extraction method for the twisted pair cable with rectangular connectors

**DOI:** 10.1371/journal.pone.0205072

**Published:** 2018-10-05

**Authors:** Wei Fang, Yongfeng Ren, Xin Liu, Xinquan Jiao, Chengqun Chu

**Affiliations:** 1 National Key Laboratory for Electronic Measurement Technology, North University of China, Taiyuan, Shanxi, China; 2 School of Electrical and Control Engineering, North University of China, Taiyuan, Shanxi, China; 3 College of Electronics and Information Engineering, Taiyuan University of Science and Technology, Taiyuan, Shanxi, China; University of Scranton, UNITED STATES

## Abstract

Cables play an important role in transmitting energy and information. In this article, in order to obtain the S-parameters of the special twisted pair cable with rectangular connectors, a de-embedding method of the transmission matrix is proposed to deal with the two adapters ends of the cable. According to the frequency-dependent *RLGC(f)* model, the cable characteristics are extracted in the frequency range of 10 MHz to 200 MHz through conventional and modified methods respectively. The frequency-domain analysis shows that the inherent capacity of modified method can decrease the errors especially due to the discontinuities of hyperbolic functions. This research supplies an advantageous modelling approach for the cable to improve its robustness against disturbances in the S-parameters measurement that cannot be decreased with the calibration procedure.

## Introduction

Twisted pair cables are widely used in various modern electronic devices and systems possessed with the characteristics of low cost, low loss, and low coupling, such as controller area network buses, communication systems, airplanes, and ships [1]. As operating frequencies increase, signal integrity analysis and channel modeling are becoming increasingly significant in high-speed digital systems. When the system frequency is in the hundreds of megahertz, or even as high as gigahertz, the cable is no longer a simple conductor, but exhibits a high-frequency effect, and behaves as a transmission line [2]. Thus, it is critical to establish an accurate frequency-dependent *RLGC*(*f*) model for cables in high-frequency range. The special cable used in this article is a twisted pair copper cable with rectangular connectors at both ends, which makes it difficult to obtain accurate RLGC parameters.

In past studies, there are many methods to extract RLGC parameters. Some of them are based on S-parameter measurements [3–8] while others are based on time-domain measurements [9–12]. In [3], Zhang et al presented three frequency-dependent *RLGC*(*f*) models, and took into account high-frequency effects, such as skin effect loss, dielectric dispersion and crosstalk. A good agreement of simulated and measured S-parameters has been achieved in the frequency domain for the three transmission lines by using the proposed frequency-dependent *RLGC*(*f*) models. In the frequency range of 300KHz to 300MHz, Papazyan et al [4] used S-parameter curve fitting to minimize error, and directly calculated the RLGC parameters from the propagation constant γ and characteristic impedance Z_C_. In [11], Kim et al used rise time of step pulse measured by the Time Domain Reflectometer (TDR) to calculateγ. For these methods, whether based on frequency domain or time domain measurements, RLGC parameters can be extracted by calculation of propagation constant γ and characteristic impedance Z_C_ and then calculated for RLGC parameters from γ and Z_C_. These methods are called as the ‘direct calculation’ methods.

In fact, these methods cannot yield accurate results because of the launch effects in calculation of Z_C_ and slight measurement errors. Especially in measuring S-parameters, since Vector Network Analyzer (VNA) can only be measured on a well-defined 50Ω coaxial interface, a reasonable fixture design or de-embedding technique is necessary. Sampath presented a method of comparing two similar transmission lines with different lengths for de-embedding, in order to obtain more accurate RLGC parameters [13]. Other de-embedding approach was utilized transmission matrix mathematics to bisect a symmetrical structure into mirrored parts which can be mathematically removed from both ends of the measured structure, leaving only the expected device under test [14–15]. This method offered the high frequency utility and accuracy of calibration methods without the needed manufacture and design precision, and provided an alternative to de-embedding techniques restricted by lumped element assumptions.

This article presents a measurement method for a cable containing rectangular connectors at both ends. Mathematically separating the adapters at both ends by using the transmission matrix, the ABCD matrix of the cable under test (CUT) is obtained. The RLGC parameters are calculated from ABCD matrix, then, optimized by particle swarm optimization (PSO) algorithm to get more accurate RLGC parameters.

The rest of this paper is organized as follows. The next section illustrates the method and principle of the test. Section 3 deduces the relevant equations of RLGC parameters extraction, and provides the improved RLGC extraction method with PSO algorithm. The simulation and experimental results are discussed in Section 4. Section 5 summarizes the most important conclusions.

## Methods

### Test method and principle

**Fig 1:**
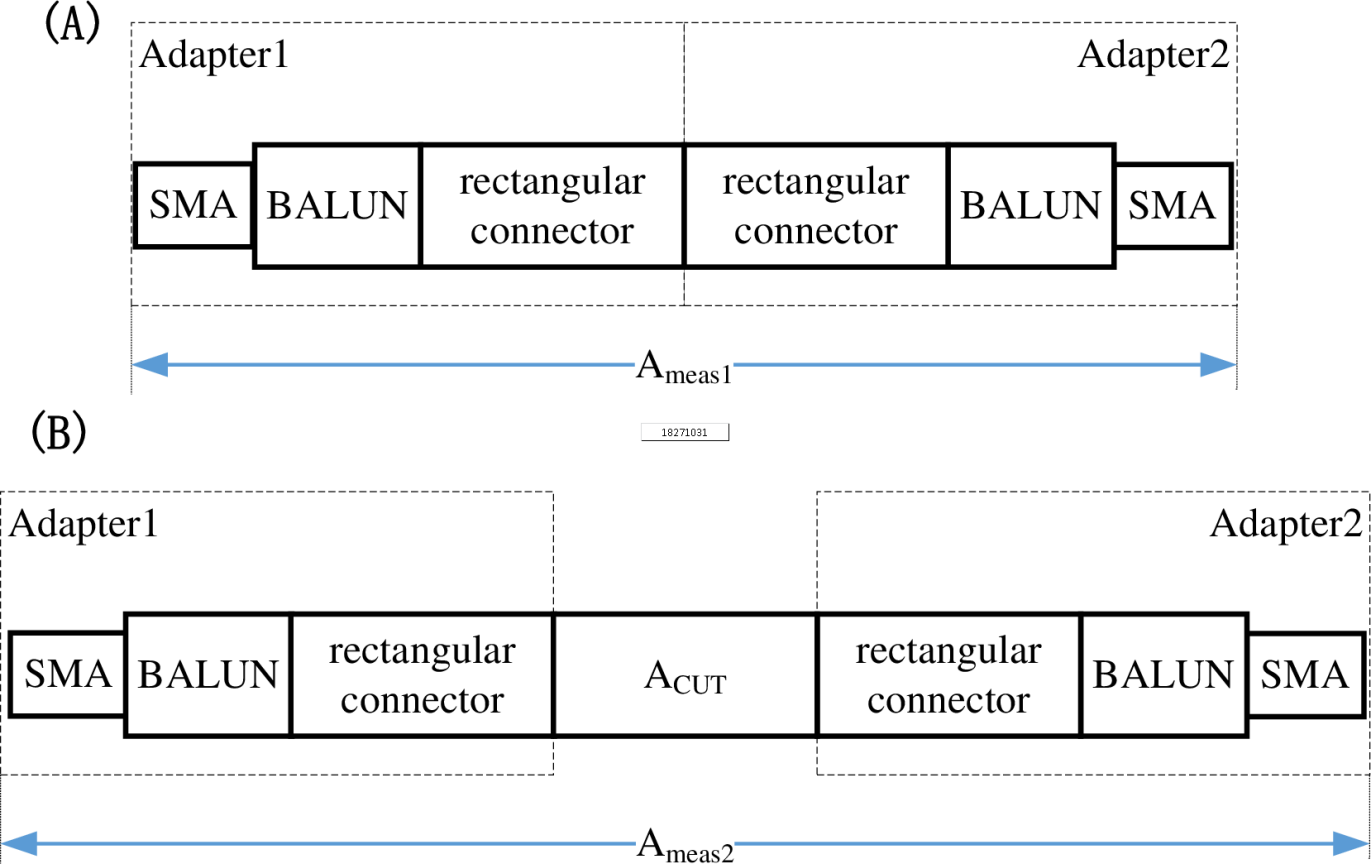
Graphical representation of measured structure, showing (A) measured structure of adapters directly connected and (B) measured structure containing embedded cable under test.

The cable studied in this article is equipped with a rectangular connector at either end to facilitate connection with other equipment or cables. Two adapters are first designed to connect with the VNA coaxial interface to measure the S-parameters of this dedicated cable accurately. The adapters are of the same structure, either made up of an SMA, a rectangular connector, a balun and necessary lines. The structures required for the bisecting de-embedding method to be utilized are shown in block scheme in Fig 1. A symmetrical structure is made up of two adapters directly connected (Fig 1A) and the desired CUT is placed between the two adapters (Fig 1B). Through mathematical conversion, the transmission matrix is used to split the symmetrical structure of the two adapters into mirrored parts which may be mathematically removed from both ends of the cable, leaving only the CUT.

**Fig 2:**
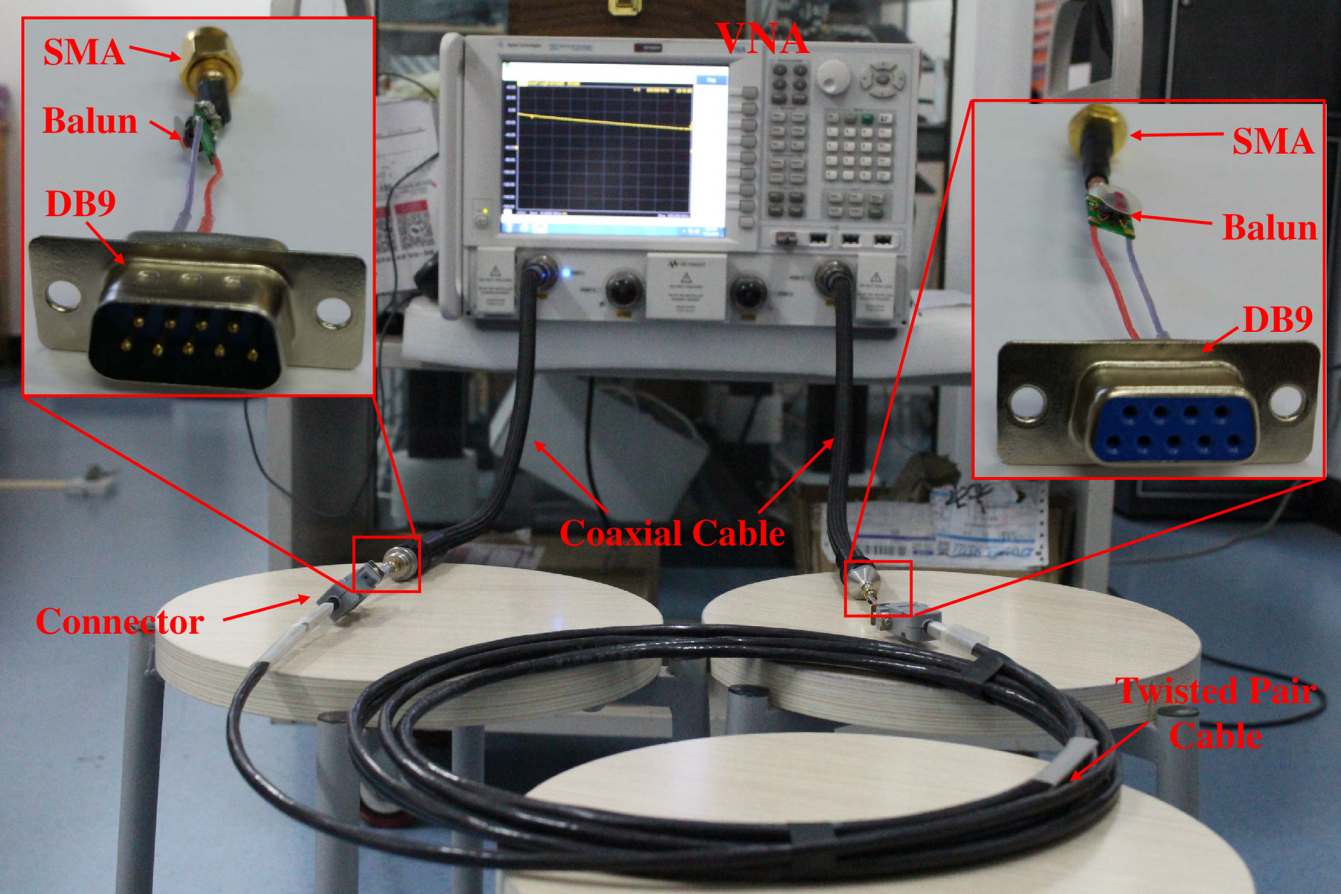
Measurement setup for S-parameters.

The measurement setup is shown in Fig 2. In order to measure the S-parameters, two 0.5 m length 85133–60016 type coaxial cables with 50 Ω characteristic impedance were attached to the ports for the connection between the VNA and the CUT. Two port calibration was used to eliminate unwanted connection effects caused by coaxial cables. These experimental studies were carried out without any extra load connection to determine the transmission line parameters. The frequency range of this experiment is 10~200 MHz, and the IF bandwidth is 10 KHz as well as the 951 points for the VNA setting.

For this work, S-parameters are transferred to ABCD matrix format in order to carry out the required network calculations, which is suitable for cascade matrices or other transmission matrices.

In order to bisect the symmetrical structure *A*_*meas*1_ composed of two adapters, it is necessary to solve the ABCD matrix equation, as shown in (1).

$$A_{meas1}=A_{1}\cdot A_{2}$$ (1)

where A_1_ and A_2_ represent ABCD matrices of adapters 1 and 2 respectively.

Following the definition presented in [14], as adapters 1 and 2 are of the same structure but with reversed ports, the ABCD matrix of adapter 2 can be represented by symmetric conjugate of the ABCD matrix corresponding to adapter 1 and defined as follows:

$$A_{2}=\bar{A_{1}}=\bar{\left[\begin{array}{cc} a & b\\ c & d \end{array}\right]}=\frac{1}{ad-bc}\left[\begin{array}{cc} d & b\\ c & a \end{array}\right]$$ (2)

where the overline indicates symmetric conjugation.

Eq (1) generally has no solution unless adapter 1 is completely symmetrical to adapter 2. In fact, there is no exact solution to (1) due to measurement errors and asymmetries in the practical structure. Hence, it can only be solved approximately. A network that is entirely made up of linear passive components, such as connectors and transmission lines, is reciprocal [16]. That means *ad* − *bc* = 1. Thus, A_1_ and A_2_ can be calculated from (1) and (2).

In practice, even though adapters 1, 2 are of the same structure, for slight measurement errors and imperfections in the physical structure, *A*_*meas*1_ is not strictly symmetric, and in general *S*_11_ ≠ *S*_22_ (the obtained data is never strictly symmetric). Although there are slight asymmetries, the proposed approach is proved to be robust. Advantage of the approach is that reasonable de-embedding can be achieved without the known impedance and other specific characteristics.

Once A_1_ and A_2_ have been obtained, A_CUT_ can be calculated simply as follows:

$$A_{CUT}=A_{1}^{-1}\cdot A_{meas2}\cdot A_{2}^{-1}$$ (3)

### Conventional RLGC extraction method

To extract accurate RLGC parameters with the measured S-parameters by the conventional method (CM), there are many difficulties. The errors relating to the discontinuities of hyperbolic functions are inevitably introduced. Generally speaking, these equations are of strict restrictions on the RLGC extraction process, such as specific length, specific frequency band, and the number of branches, etc.

RLGC parameters in CM are usually extracted by transferring measured S-parameter matrix to Z-parameter matrix, as shown in (4).

$$Z=\left[\begin{array}{cc} Z_{11} & Z_{12}\\ Z_{21} & Z_{22} \end{array}\right]=Z_{0}\left(I+S\right){\left(I-S\right)}^{-1}$$ (4)

where I is the identity matrix, S is S-parameter matrix, and Z_0_ is the reference impedance matrix (50Ω). Then, S-parameter matrix is converted to the T-transmission matrix as shown in (5).

$$T=\left[\begin{array}{cc} T_{11} & T_{12}\\ T_{21} & T_{22} \end{array}\right]=\left[\begin{array}{cc} Z_{11}Z_{21}^{-1} & Z_{11}Z_{21}^{-1}Z_{22}-Z_{12}\\ Z_{21}^{-1} & Z_{21}^{-1}Z_{22} \end{array}\right]$$ (5)

where Z is Z-parameter matrix. T-transmission matrix of the CUT (5) refers to ABCD parameters as in (6).

$$T=\left[\begin{array}{cc} A & B\\ C & D \end{array}\right]=\left[\begin{array}{cc} Z_{c}\cosh\left(\gamma l\right)Z_{c}^{-1} & Z_{c}\sinh\left(\gamma l\right)\\ \sinh\left(\gamma l\right)Z_{c}^{-1} & \cosh\left(\gamma l\right) \end{array}\right]$$ (6)

where γ is propagation constant, Z_C_ is characteristic impedance, and l is length of the transmission line.

The propagation constantγand characteristic impedance Z_C_ are calculated as follows [17]:

$$\gamma =\sqrt{\left(R\left(f\right)+j2\pi fL\left(f\right)\right)\left(G\left(f\right)+j2\pi fC\left(f\right)\right)}$$ (7)

$$Z_{c}=\sqrt{\frac{R\left(f\right)+j2\pi fL\left(f\right)}{G\left(f\right)+j2\pi fC\left(f\right)}}$$ (8)

RLGC parameters can be obtained by the formula (4), (5) and (6) for each frequency point as in (9) [18].

R(f)=Re(Zcγ)G(f)=Re(γZc−1)L(f)=Im(Zcγ)/(2πf)C(f)=Im(γZc−1)/(2πf) (9)

where Re () and Im () represent for the real and imaginary part of their items in (9), respectively.

### Modified RLGC extraction method

In contrast to CM, a modified method (MM) which extracts RLGC parameters from the measured S-parameters is shown in (10) [19] to avoid discontinuities caused by hyperbolic functions. Especially, the above limitations of the conventional methods are ignored in the modified method, such as the specific line lengths, specific frequencies and the number of branches, etc. Since this method can decrease the discontinuities due to the hyperbolic functions, the model exhibits more effective and accurate in a larger frequency band.

$$\{\begin{array}{l} R\left(f\right)=R_{1}+R_{2}\sqrt{f}\\ L\left(f\right)=L_{1}+R_{2}/\left(2\pi \sqrt{f}\right)\\ G\left(f\right)=G_{1}+G_{2}f\\ C\left(f\right)=C_{1} \end{array}$$ (10)

where R_1_, R_2_, L_1_, G_1_, G_2_ and C_1_ are unknown constants to be estimated. R_1_ is the DC resistance, R_2_ refers to the skin effect loss, L_1_ is the high frequency inductance (usually constant), G_1_ is shunt current caused by free electrons in imperfect dielectric, G_2_ refers to the power loss on account of dielectric polarization, and C_1_ is the geometric-related capacitance constant.

Due to the discontinuities of the hyperbolic functions, the conversion between the RLGC values and S-parameters in the objective function suffers a lot from non-linear, which should be improved by a non-linear optimization algorithm. So, many researchers used optimization algorithms such as genetic algorithm to search global minimum [20]. In this research, we choose the Particle Swarm Optimization (PSO) algorithm [21], for it does not need to pick up a reasonable initial value in a given search range of parameters, which is easy to implement and to find the global minimum effectively. The purpose of the objective function is to minimize the errors between the estimated and measured S-parameters, as shown in (11).

Fobj=1N∑i=1N|Re(Sjkm(fi)−Sjke(fi))|+1N∑i=1N|Im(Sjkm(fi)−Sjke(fi))|j,k∈[1,2] (11)

where $S_{jk}^{m}\left(f_{i}\right)$ and $S_{jk}^{e}\left(f_{i}\right)$ are measured and estimated S-parameters at i^th^ frequency, respectively.

The estimated S-parameters are calculated from the RLGC parameters by known equations. Firstly, the propagation constants γ and characteristic impedances Z_C_ are calculated from the RLGC parameters by (7) and (8), respectively. Secondly, the ABCD matrix is obtained withγand Z_C_ parameters, while *l* = 6*m* line length as in (6). Finally, the estimated S-parameters (S^e^) are obtained from ABCD matrix as in (12) [22].

$$\begin{array}{l} S^{e}=\left[\begin{array}{cc} S_{11}^{e} & S_{12}^{e}\\ S_{21}^{e} & S_{22}^{e} \end{array}\right]=\left[\begin{array}{cc} \frac{AZ_{0}+B-CZ_{0}^{2}-DZ_{0}}{\nabla } & \frac{2\left(AD-BC\right)Z_{0}}{\nabla }\\ \frac{2Z_{0}}{\nabla } & \frac{-AZ_{0}+B-CZ_{0}^{2}+DZ_{0}}{\nabla } \end{array}\right]\\ \nabla =AZ_{0}+B+CZ_{0}^{2}+DZ_{0} \end{array}$$ (12)

**Fig 3:**
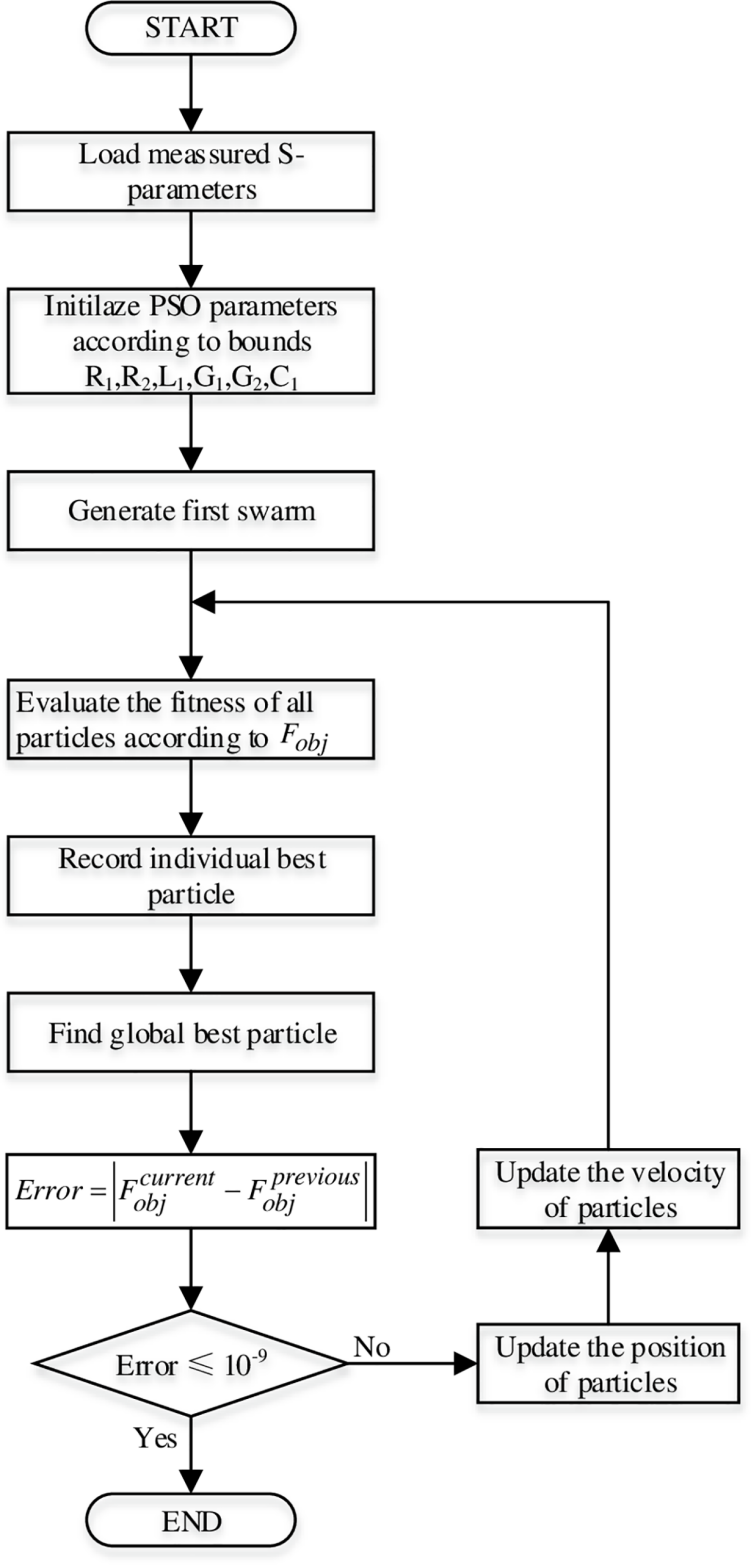
Optimization process flowchart of the model parameters extraction.

The flowchart of PSO parameter estimation process is given as in Fig 3.

Search ranges for multiple parameters used in PSO are shown in Table 1. Although PSO does not require any boundaries, limiting the search area of well-defined boundaries, it can increase the opportunity to find the global minimum with fewer iterations. There are many criteria to complete PSO iterations, such as target value of objective function, maximum iteration number, and relative error between two successive iterations. Once any of these criteria has been met, the algorithm stops immediately.

**Table 1:** Upper and lower bounds of the parameters used in the PSO.

*R*_1_(Ω/*m*)	$R_{2}\left(\Omega /m\cdot \sqrt{Hz}\right)$	*L*_1_(*H*/*m*)	*G*_1_(*S*/*m*)	*G*_2_(Ω/*m* ⋅ *Hz*)	*C*_1_(*F*/*m*)
0~10	0~1	0~10^−5^	0~1	10^−11^~10^−8^	10^−11^~10^−8^

## Results and discussion

The experimental data were obtained by using a calibrated Keysight VNA and corresponding adapters. The cable for test is a twisted pair copper with length of 6-meter. The type number is CAT5E, which has a rectangular connector at each end of cable. MATLAB software was used to carry out all the simulation and RLGC parameters of the cable were extracted respectively by CM and MM.

**Fig 4:**
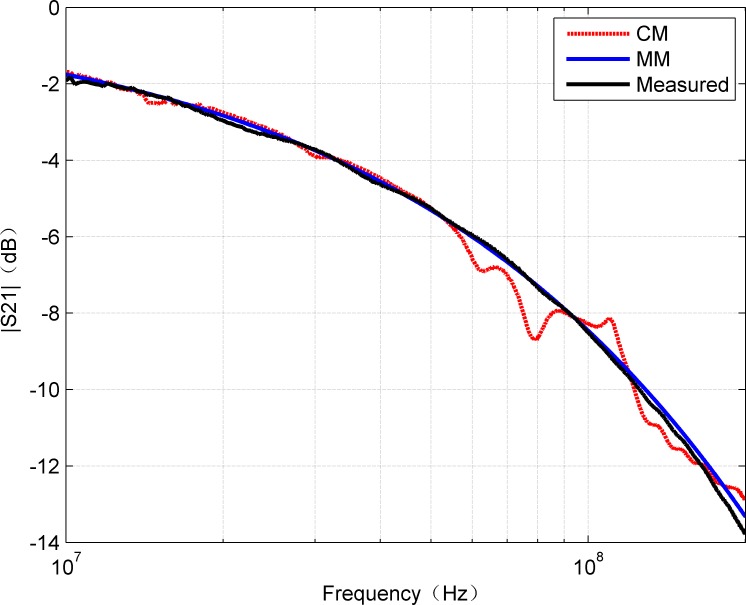
Comparison of amplitude of measured S_21_ with the results of CM and MM.

**Fig 5:**
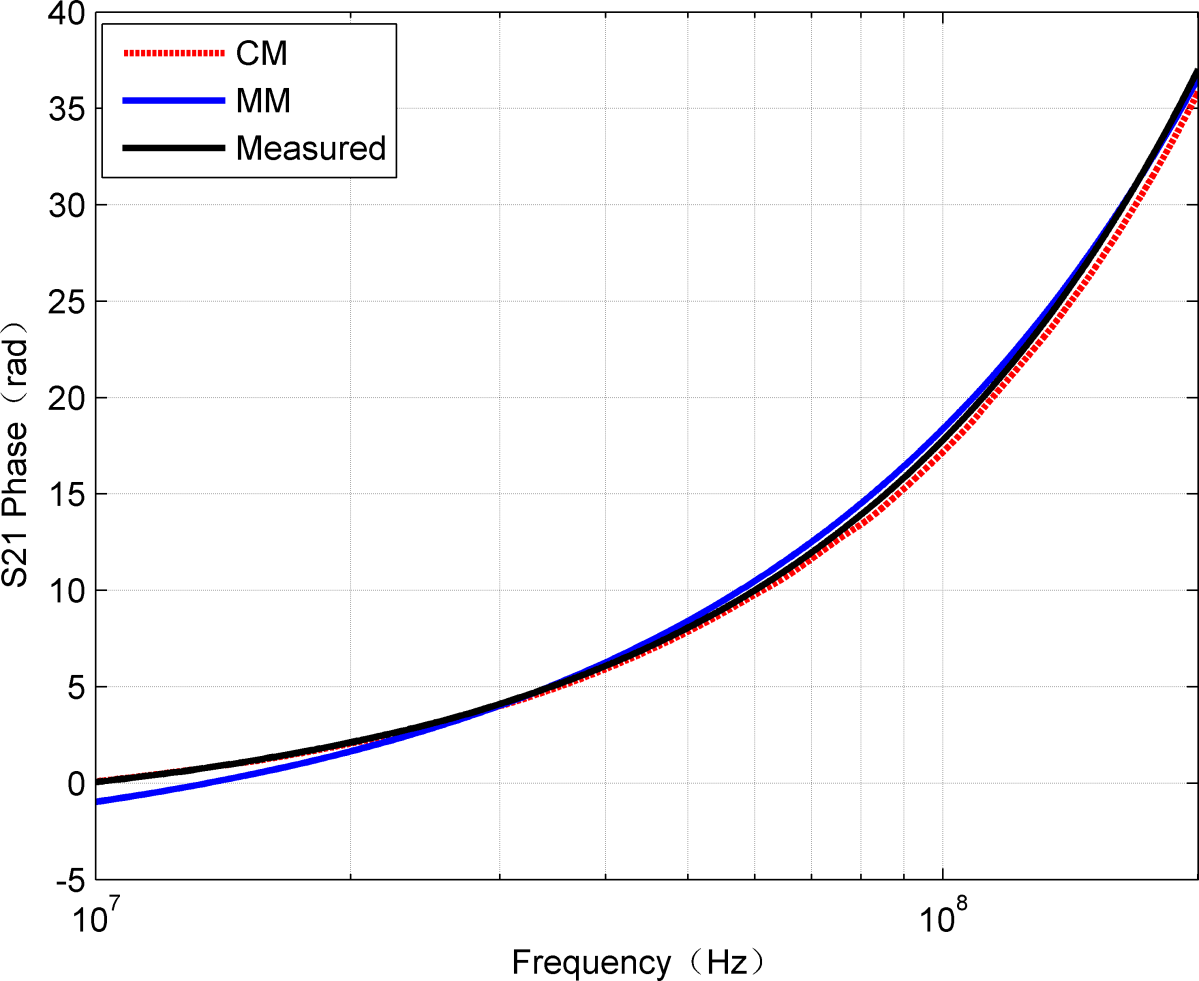
Comparison of phase of measured S_21_ with the results of CM and MM.

Since S_21_ represents the transfer function of the system, the calculated S_21_ by CM and MM methods was compared with the measured result, which is shown in Fig 4. It can be concluded from the Fig 4 that the amplitude of S_21_ decreases with the increase of frequency, and the result of MM keeps good agreement with the measured value. Essentially, the communication channel path loss increases with frequency. It can also be found that above 50MHz, the MM result outperforms CM. On the other hand, S-parameters are usually evaluated in the form of their amplitude and phase graphs. Although the calculation of S_21_ is straightforward, the phase constant is periodic and can only be uniquely determined by the function of “unwrapping”. Therefore, the visual comparisons of the measured data with the two methods were made according to the unwrapped phase graph of S_21_ as shown in Fig 5. All the three methods almost have the same results in the phase of S_21_.

**Fig 6:**
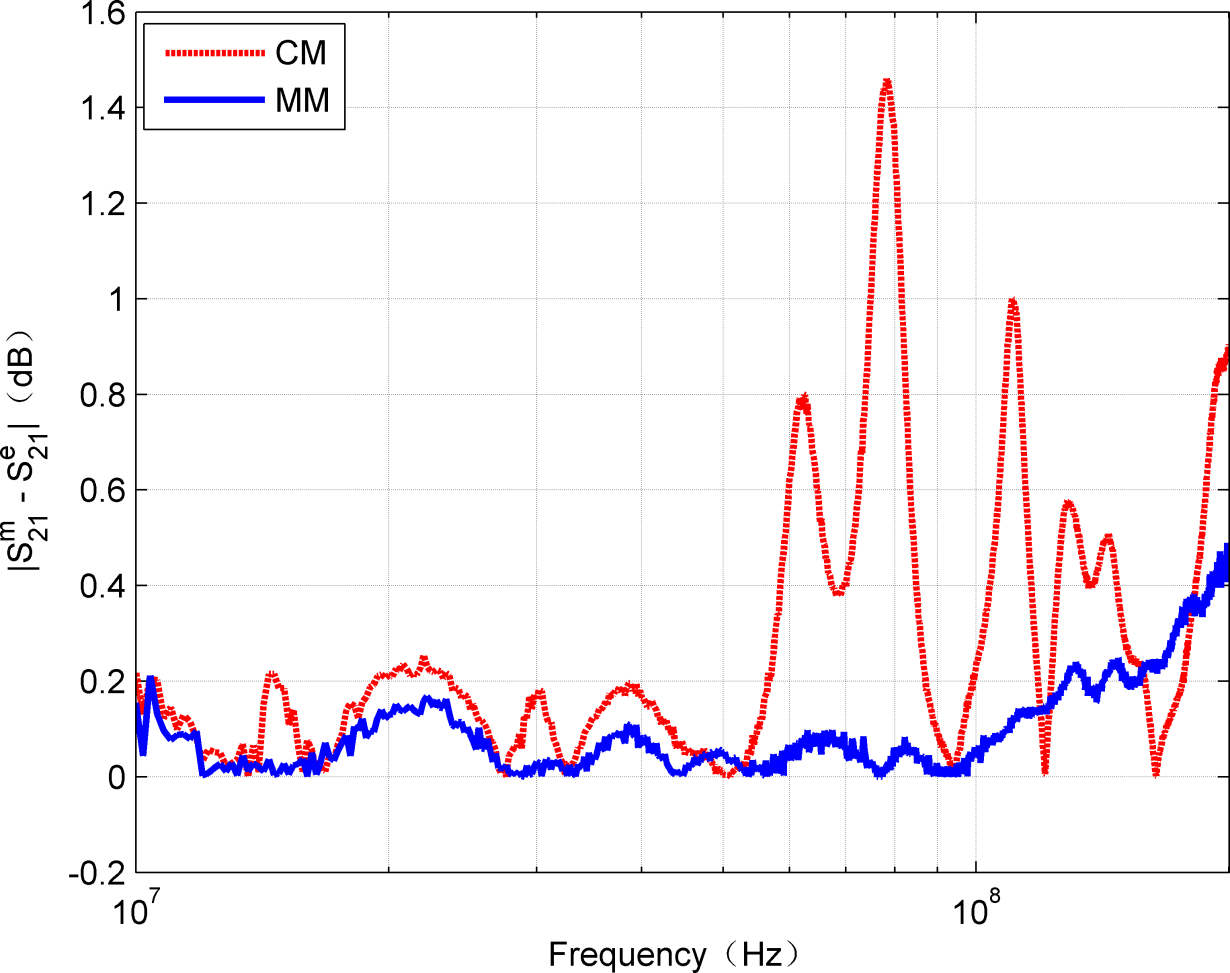
Comparison of absolute amplitude error of estimated and measured S_21_.

**Fig 7:**
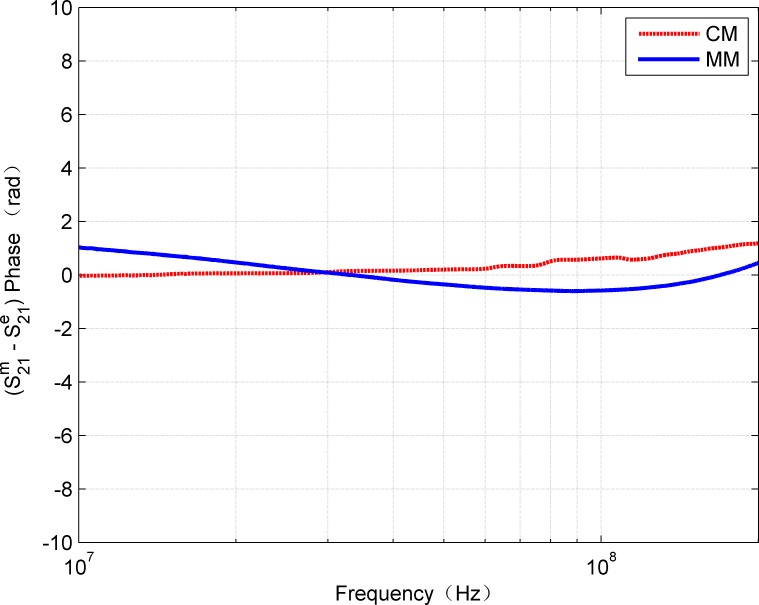
Comparison of absolute phase error of estimated and measured S_21_.

In addition, for complete comparison, Fig 6 and Fig 7 show the errors of amplitude and phase for calculated and measured S_21_, respectively. The curves in Fig 6 confirm that MM gives smaller amplitude errors than CM. The mean of absolute error of CM is 0.3666 while MM is 0.1469. It can be observed that, the error is within 0.5 dB in the frequency range of 0-200MHz. However, due to the discontinuities of the hyperbolic functions, the amplitude error curve of CM shows some large fluctuations. In fact, above 100 MHz, the effects of inhomogeneity on account of the imperfect structures of the twisted pair cable become more prominent and inconsistent than the ideal model (e.g., the proposed model). When the frequency is up to 200MHz, it was noted the maximum error calculated by the MM is about 0.5dB, which is reasonable for many applications, while the maximum error calculated by CM is approximate 1.45dB. It can be seen that CM is also sensitive to the measurement method except the influence caused by the discontinuities of the hyperbolic functions. Moreover, it can be seen from Fig 7 that the phase values calculated by CM and MM are very close to the measured values. In general, the R-square value was used as quantitative error measure. Table 2 contains the R-square values for amplitude and phase of S_21_ by CM and MM, respectively. Because the R-square value of 1 means an exact match, the obtained error values show that the model results can be considered as a very close estimation. Thus, it can be predicted that the proposed MM can be applied within the range of 0-200MHz with relatively high reliability.

**Table 2:** The R-square values of amplitude and phase of S_21_ by CM and MM.

	Amplitude of S_21_	Phase of S_21_
CM	0.9526	0.9368
MM	0.9703	0.9801

**Fig 8:**
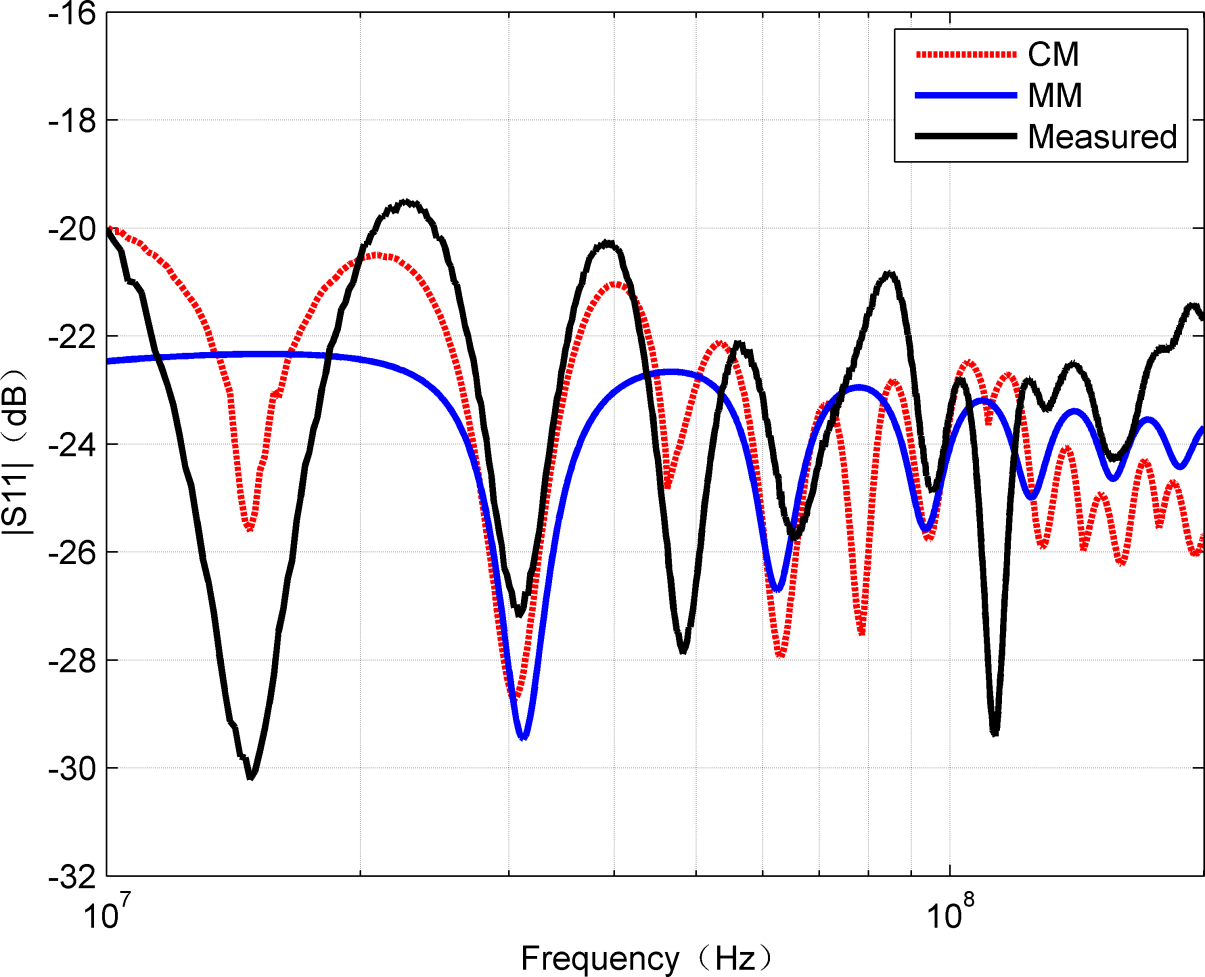
Comparison of amplitude of measured S_11_ with the results of CM and MM.

For the sake of completion, the amplitude of S_11_ estimated by CM and MM is compared with the measured results as shown in Fig 8. It can be observed from Fig 8 that the amplitude of S_11_ is below -19.5dB, which indicates that the return loss is small and cannot impact the signal transmission.

**Fig 9:**
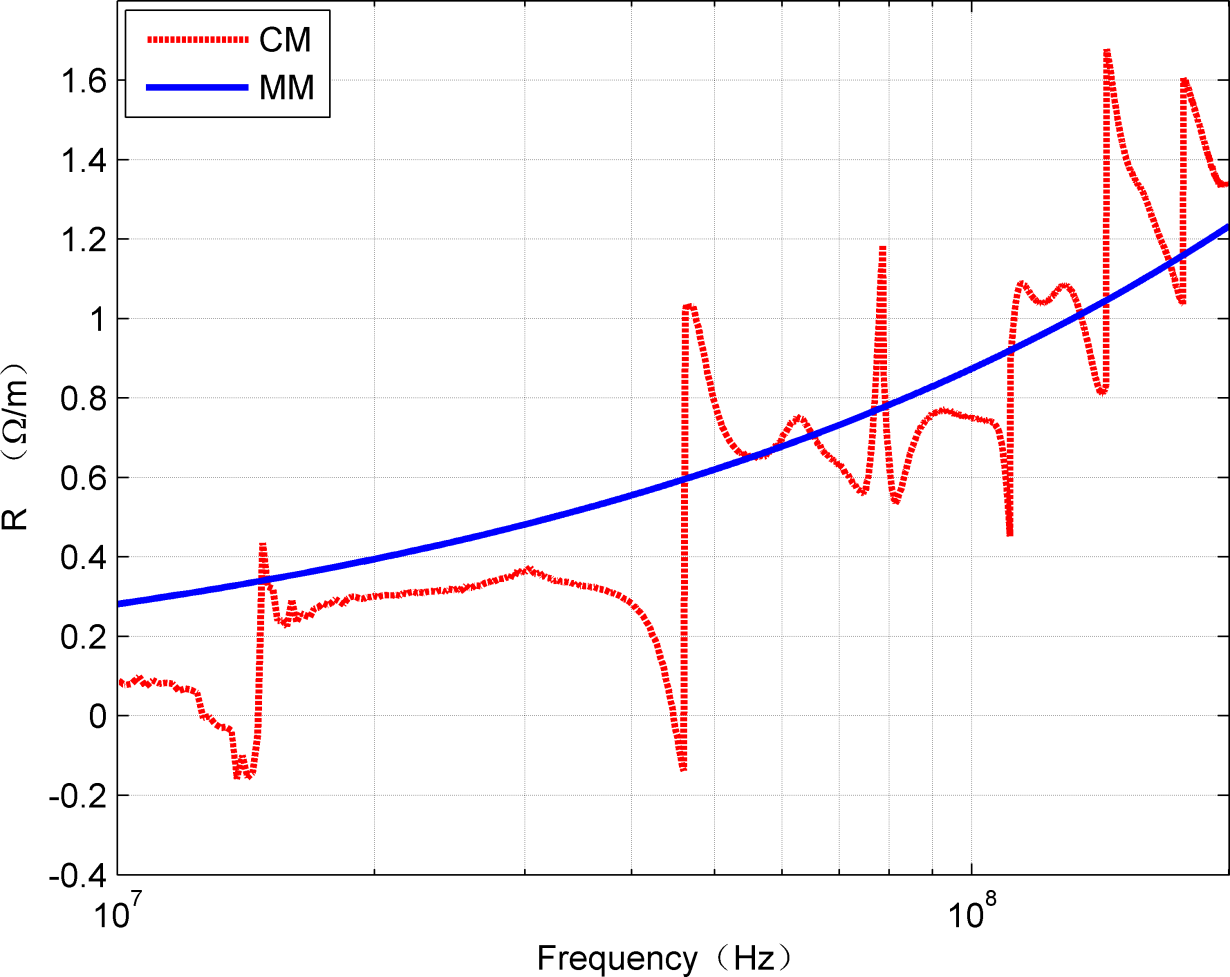
Comparison of *R*(*f*) estimated from CM and MM.

**Fig 10:**
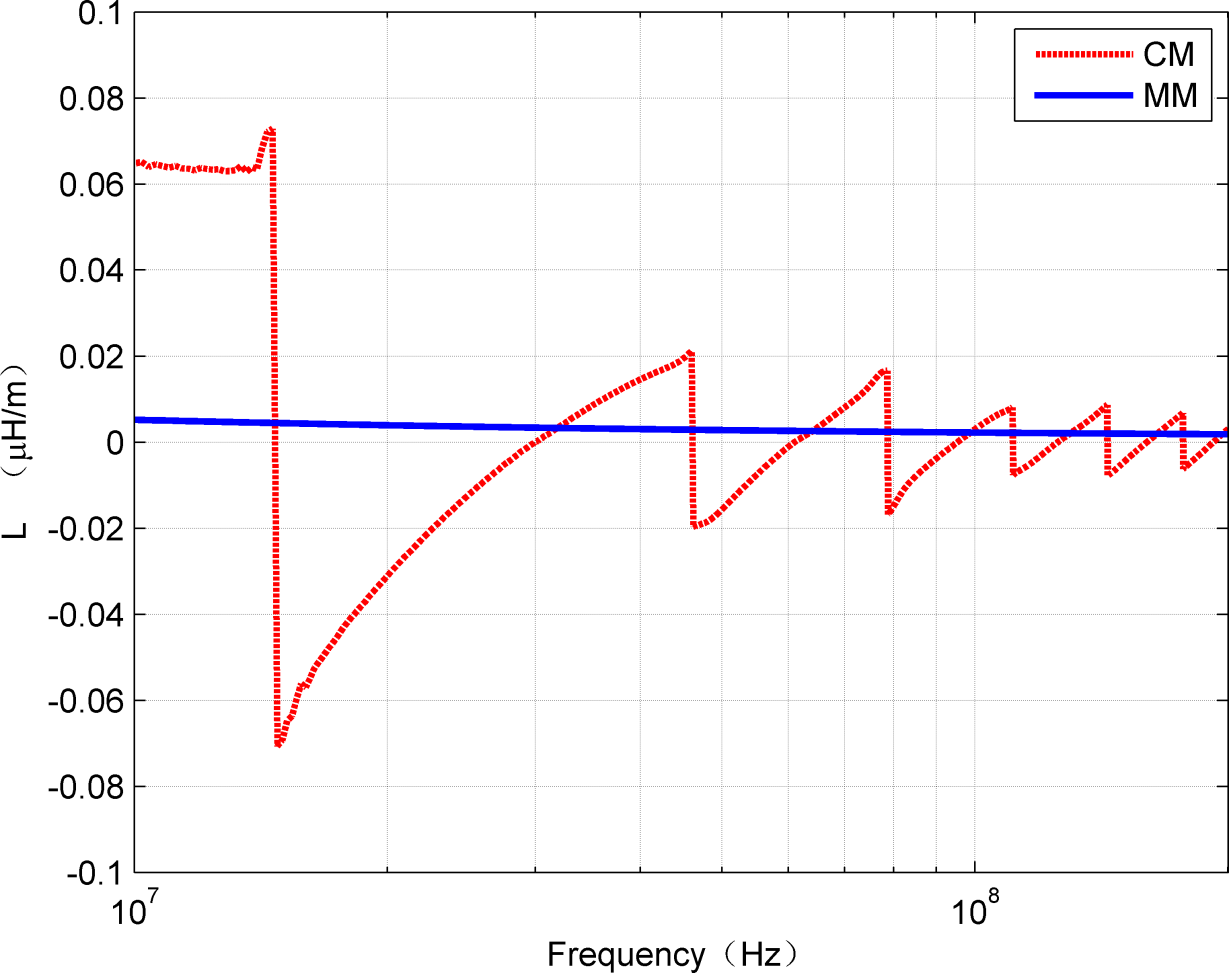
Comparison of *L*(*f*) estimated from CM and MM.

Fig 9 and Fig 10 show the R and L values calculated from CM and MM, respectively. The mean value of R calculated from CM and MM was 0.8647 Ω/m and 0.8576 Ω/m while the L was about 0.00233 μH/m and 0.00245 μH/m.

Although the mean value of R and L obtained by the both methods is similar, it can be clearly seen from Fig 9 and Fig 10 that both the curves of R and L obtained by CM have strong fluctuations. These fluctuations are mainly caused by the discontinuities of the hyperbolic functions, and they produce negative values that cannot be achieved in physical systems.

**Fig 11:**
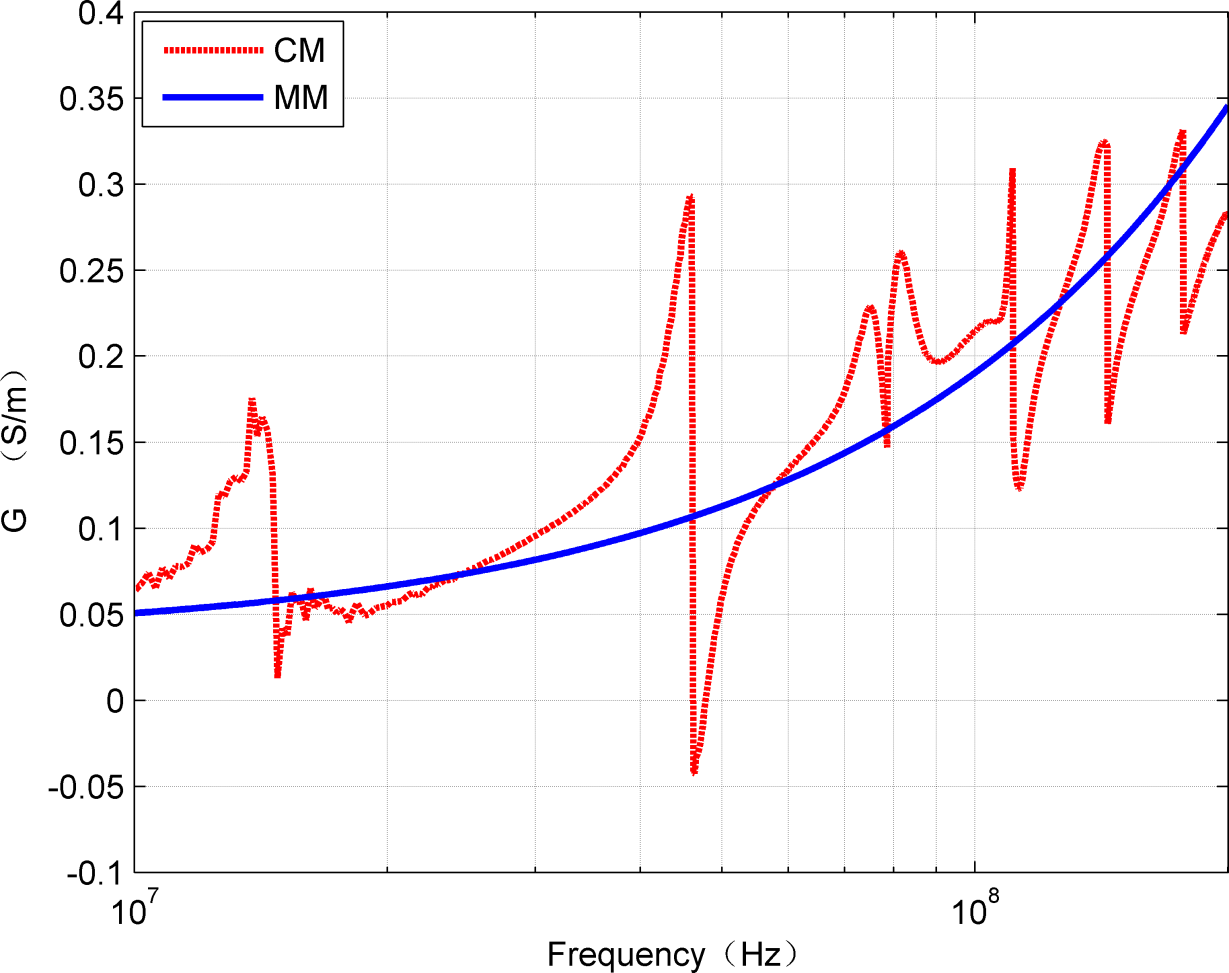
Comparison of *G*(*f*) estimated from CM and MM.

**Fig 12:**
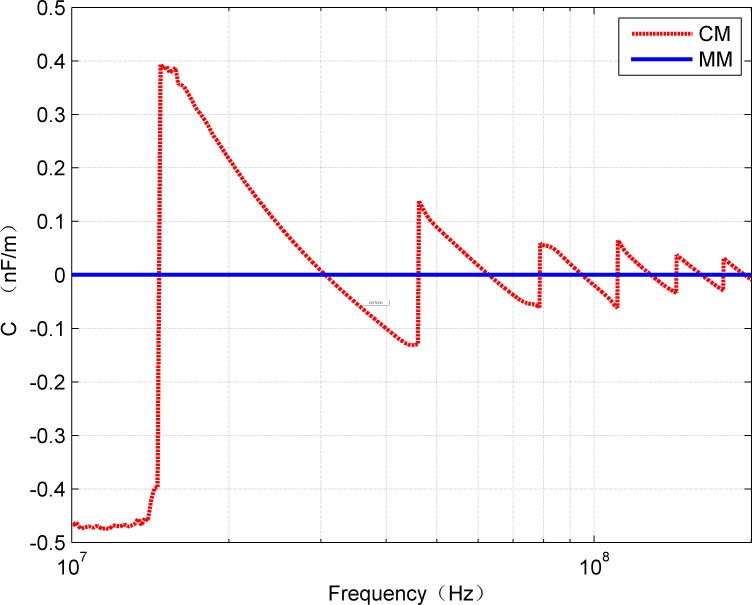
Comparison of *C*(*f*) estimated from CM and MM.

The Comparative results of the G and C values obtained by the two methods are shown in Fig 11 and Fig 12, respectively. It can be seen that the cases mentioned for R and L also appear in the G and C values.

**Fig 13:**
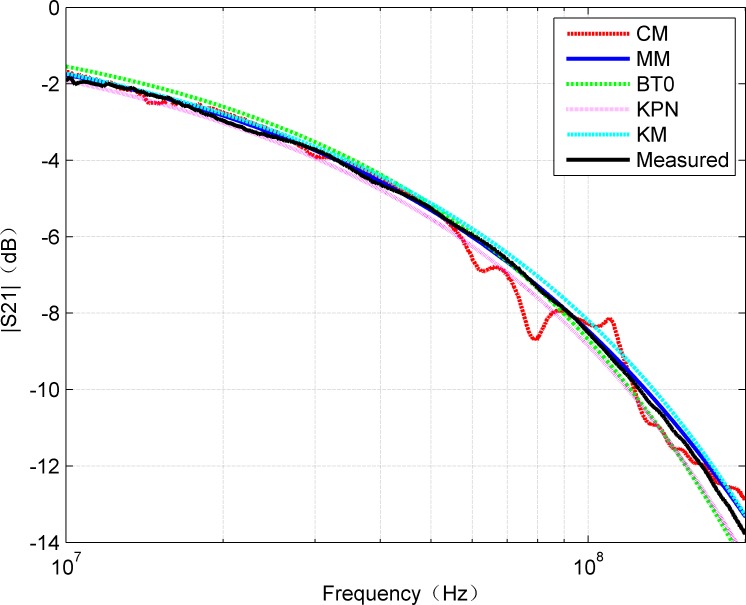
Comparison of amplitude of obtained S_21_ for each model.

**Fig 14:**
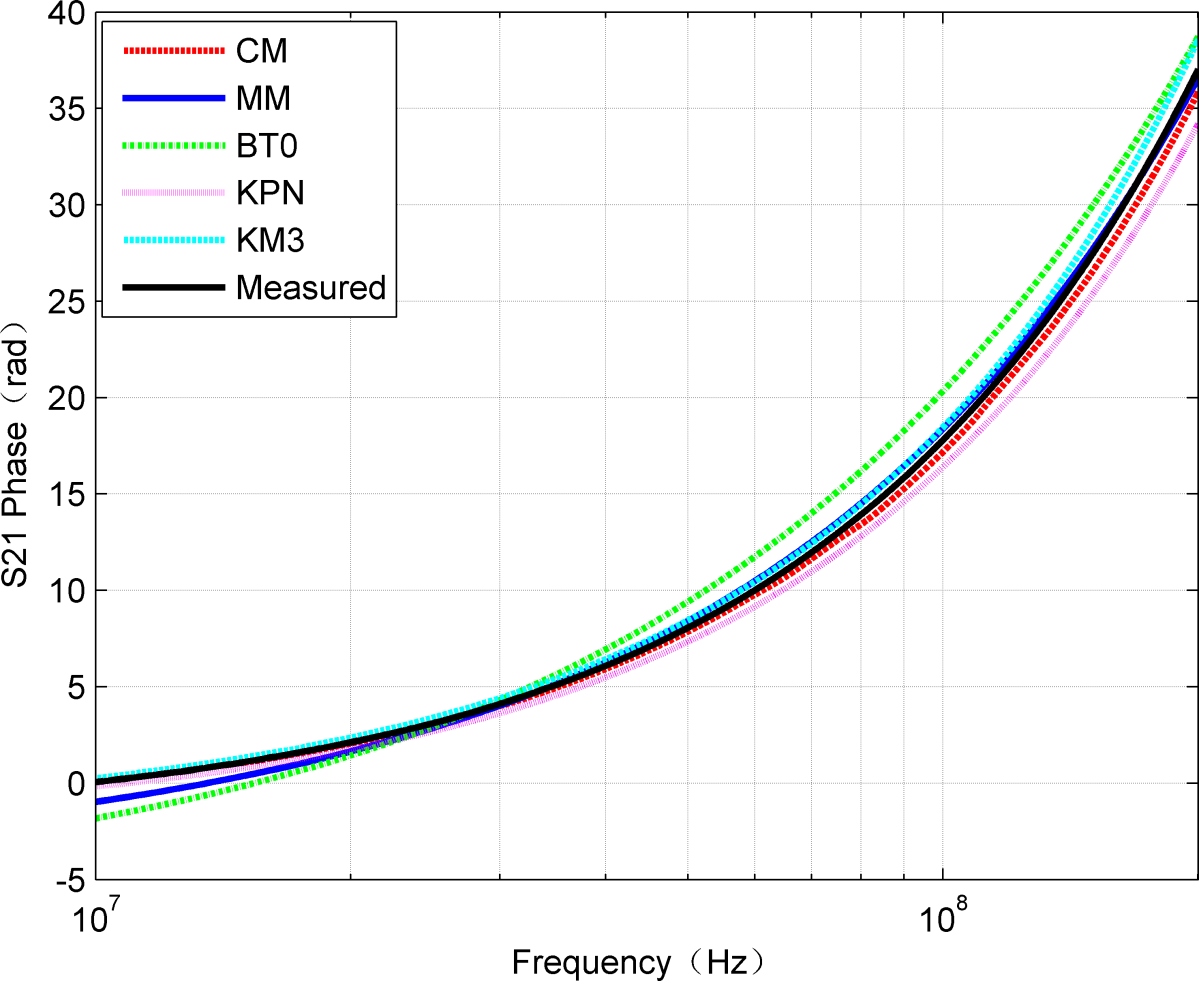
Comparison of phase of obtained S_21_ for each model.

At present, there are three typical existing models for cable characteristic parameter extraction methods. The British Telecommunications presented the empirical model (usually referred as BT0 model [23]) which was focused in modeling twisted pair copper cables by their primary parameters, RLGC. The Royal PTT Netherland presented a novel transmission line model (referred as KPN model [24]) which described the skin effect in a way that was closer related to the underlying physics. Acatauassu [25] proposed modified K-parameter model (referred as KM model) based on Chen's model. The mathematical approach of this model was focused on a function that took into account the cable gauge, the cable length and its propagation constant for a given frequency range. The amplitude and phase of S_21_ obtained by three cable models are compared comprehensively with results of the CM and MM as shown in Fig 13 and Fig 14.

**Fig 15:**
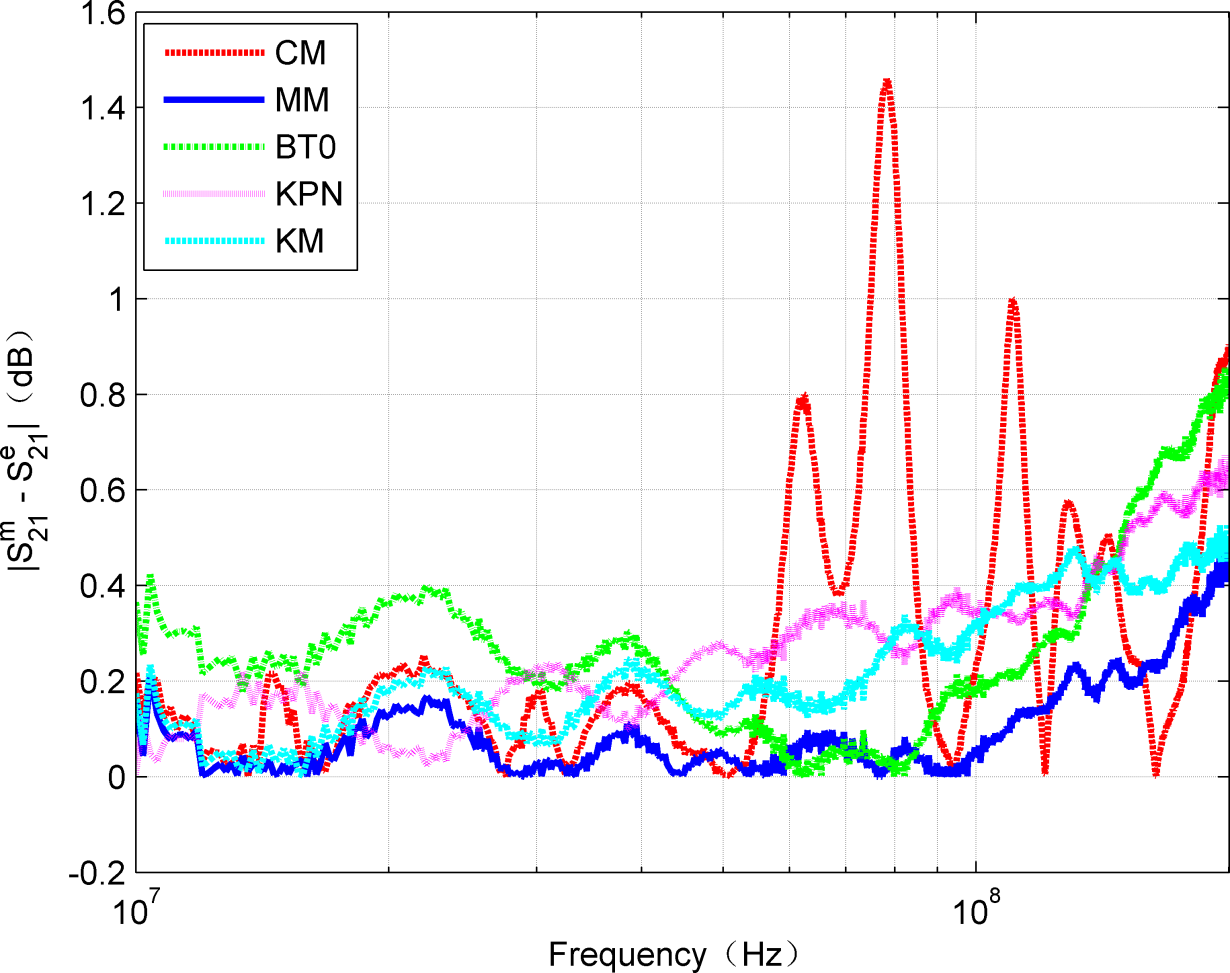
Comparison of absolute amplitude error of measured and estimated for each model.

**Fig 16:**
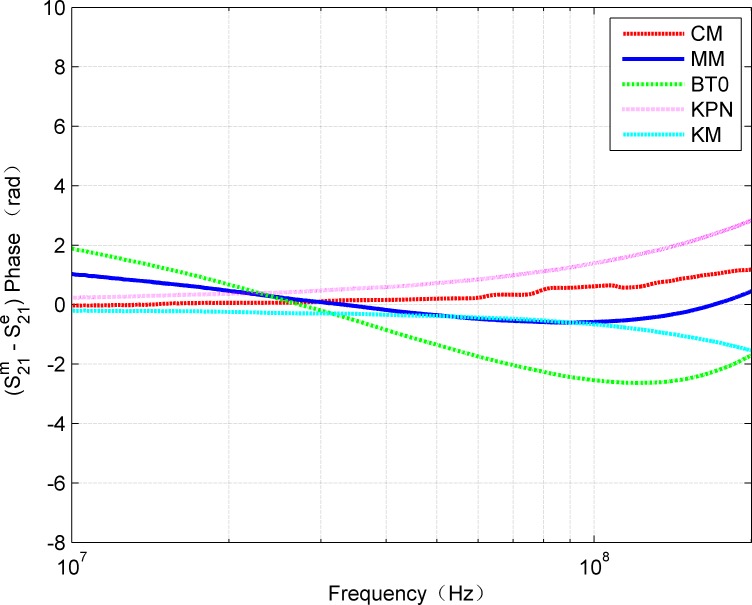
Comparison of absolute phase error of measured and estimated for each model.

It can be seen from Fig 13 and Fig 14 that the amplitude and phase curves of S_21_ obtained by each cable model keep good agreement with the measured results. The proposed MM provides the most accurate approximation for the amplitude as well as the phase characteristic. Fig 15 and Fig 16 illustrate that the absolute error curves of amplitude and phase of S_21_ are obtained by each model.

Obviously, the presented MM outperforms other models for both amplitude and phase.

## Conclusions

In practical applications, it is critical to extract RLGC parameters for cables in high-frequency range. In general, the cable is equipped with connectors at both ends, which makes it difficult to obtain accurate RLGC parameters. In this research, the S-parameters of a common twisted pair cable with connectors at both ends is measured with VNA and adapters. The two adapters are removed mathematically by transmission matrix to leave only CUT.

This study presents CM and MM to prove the feasibility of two cable characteristic parameter extraction methods. The comparisons of experimental results by two methods show that the intrinsic ability of MM can decrease the slight errors caused by the measurement faults. Although the RLGC parameters obtained from the two approaches are approximately similar, the MM results seem to be more practical and avoid negative RLGC values. During the S-parameter measurements, calibration techniques can partially minimize the slight errors existing in the process of measurements. Nevertheless, the remaining errors after calibration may still cause some mistakes in the parameter extraction procedure of the CM. The MM is capable of dealing with the remaining disturbances. For CAT5E cable, the accuracy of amplitude and phase of S_21_, which is obtained by proposed MM, are better compared with the BT0 model, KPN model, KM model, and CM. Therefore, the MM is more accurate and effective in the aspect of cable characteristic parameter extraction, especially for the cable the connectors.

In the future, the MM can be further improved by introducing some new parameters such as cable diameter, shielding material and environment temperature, etc. In addition, in order to increase the data rate, the frequency range can be enlarged to show the possibility of copper cable channels.

## Supporting information

S1 Table Original measured data. (XLSX)

S2 Table Relevant data underlying the findings described in figs 4–8. (XLSX)

S3 Table Relevant data underlying the findings described in figs 9–12. (XLSX)

S4 Table Relevant data underlying the findings described in figs 13–16. (XLSX)
